# Congenital absence of the long head of the biceps tendon: a case report

**DOI:** 10.1093/jscr/rjag571

**Published:** 2026-07-10

**Authors:** Samuel I Fuller, Taner Bay, Ellen Lutnick, Thomas Listopadzki, Michael Rauh

**Affiliations:** Department of Orthopaedic Surgery and Sports Medicine, University at Buffalo, Jacobs School of Medicine and Biomedical Sciences, 955 Main Street, Buffalo, NY 14203, United States; Department of Orthopaedic Surgery and Sports Medicine, University at Buffalo, Jacobs School of Medicine and Biomedical Sciences, 955 Main Street, Buffalo, NY 14203, United States; Department of Orthopaedic Surgery and Sports Medicine, University at Buffalo, Jacobs School of Medicine and Biomedical Sciences, 955 Main Street, Buffalo, NY 14203, United States; Department of Orthopaedic Surgery and Sports Medicine, University at Buffalo, Jacobs School of Medicine and Biomedical Sciences, 955 Main Street, Buffalo, NY 14203, United States; Department of Orthopaedic Surgery and Sports Medicine, University at Buffalo, Jacobs School of Medicine and Biomedical Sciences, 955 Main Street, Buffalo, NY 14203, United States

**Keywords:** congenital long head of biceps tendon, absence of long head of biceps tendon, long head of biceps tendon, LHBT, biceps tendon

## Abstract

This report outlines the case of a 21-year-old male, found to have a congenital absence of the long head of the biceps tendon (LHBT) following trauma, only the third reported case in this setting without a prior history of shoulder pain or instability and is one of the few cases of congenitally absent LHBT in which the condition was initially detected on magnetic resonance imaging (MRI) prior to arthroscopy. Congenitally absent LHBT may not always present with prior shoulder pain or instability; evidence of abnormality at the bicipital groove or the LHBT on MRI prior to confirmation with diagnostic arthroscopy may aid in diagnosis.

## Introduction

Congenital absence of the long head of the biceps tendon (LHBT) is rare [1–3], often incidentally discovered during imaging or surgical procedures. Previous reports have identified a wide range of clinical presentations, making its significance difficult to discern. Most manifest with shoulder pain and instability [2], with speculation on the role of the LHBT in shoulder stability [4, 5].

This case presents a 21-year-old male with a congenital absence of the LHBT evaluated for shoulder pain after a dirt bike accident, with no prior history of shoulder instability or injuries. Imaging identified a poorly visualized LHBT, with absence confirmed during arthroscopy.

## Case

A 21-year-old right-hand-dominant male presented to an orthopaedic urgent care 1 day after crashing his dirt bike, landing onto his right shoulder, with no prior injuries to the shoulder.

Examination revealed tenderness to palpation over the greater and lesser tuberosity. He had full forward flexion with pain, and limitation in internal rotation, external rotation, and abduction compared to his contralateral extremity. Strength was full. Rotator cuff testing was notable for a positive Jobe test but was otherwise unremarkable. Plain radiographs demonstrated no significant findings (Fig. 1).

**Figure 1:**
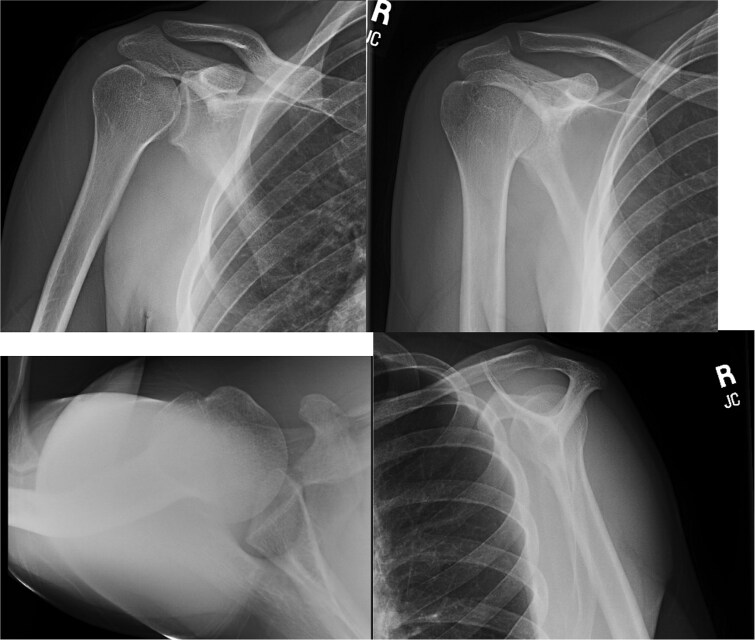
Plain radiographs of patient right shoulder on initial presentation.

Magnetic resonance imaging (MRI) performed to evaluate for rotator cuff pathology revealed increased signal interpreted as a bursal sided intermediate-high grade partial thickness tear of the anterior supraspinatus (Fig. 2). The long head of the biceps tendon was poorly visualized with a questionable, thin string of residual tendon within the bicipital groove (Fig. 3).

**Figure 2:**
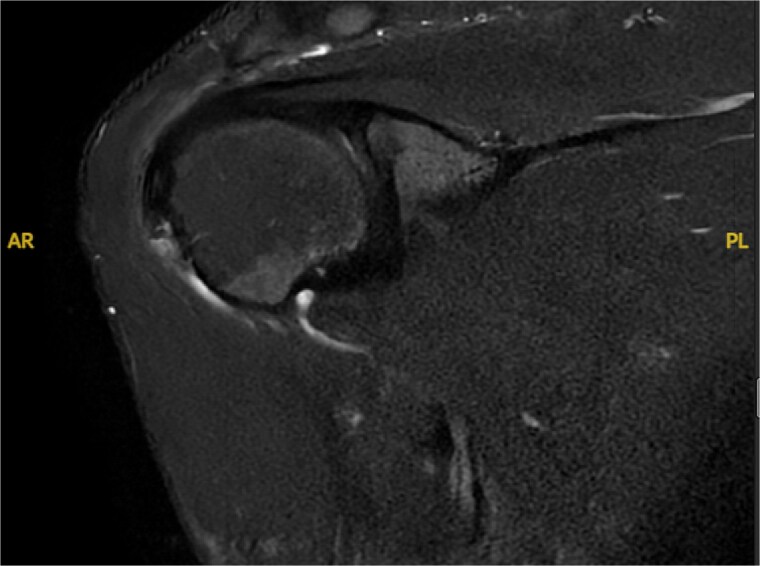
T2 coronal MRI demonstrating increased signal intensity in supraspinatus interpreted as possible bursal sided rotator cuff tear.

**Figure 3:**
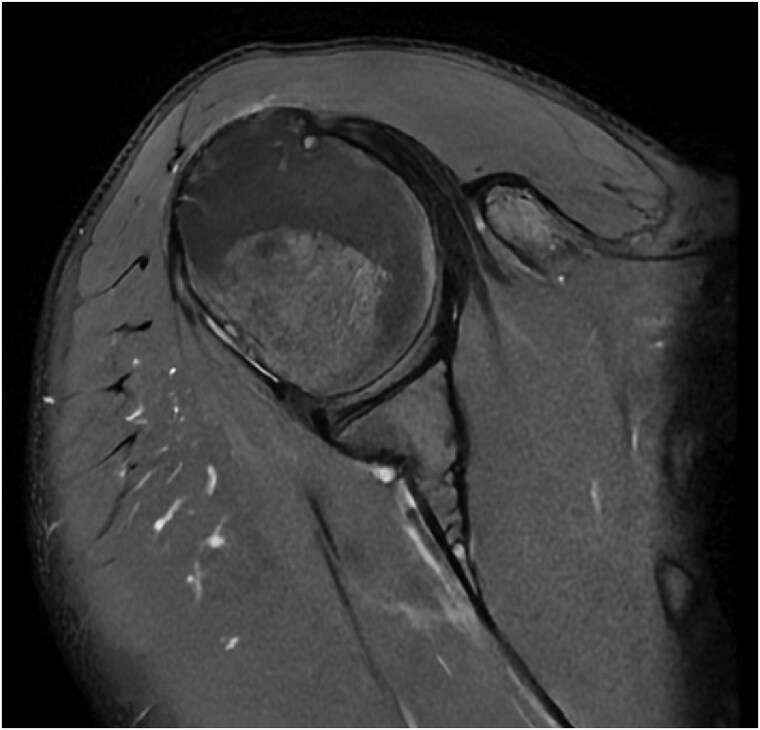
T2 axial MRI demonstrating questionable thin string of residual tendon within the bicipital groove.

Diagnostic arthroscopy was discussed and agreed upon by the treating surgeon and the patient. A standard posterior portal of the glenohumeral joint revealed a completely absent biceps tendon with no evidence of attachment at the superior labrum or surrounding synovitis (Fig. 4). The anterior portion of the cuff also revealed a congenitally absent bicipital groove (Fig. 5). Examination of the undersurface of the supraspinatus revealed no clear evidence of tearing. The subacromial space revealed extensive bursitis, a large anterior lateral spur, and partial-thickness abrasion of the anterior portion of the rotator cuff without high-grade partial or full-thickness tears. The patient underwent subacromial decompression and acromioplasty, was placed in a sling and made weight-bearing as tolerated.

**Figure 4:**
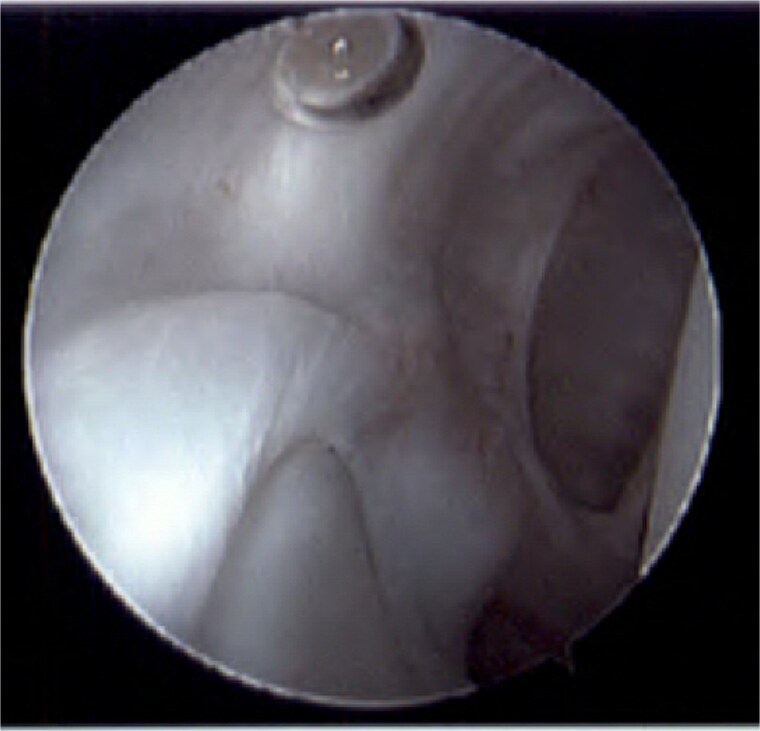
Intra-operative picture demonstrating absent biceps attachment.

**Figure 5:**
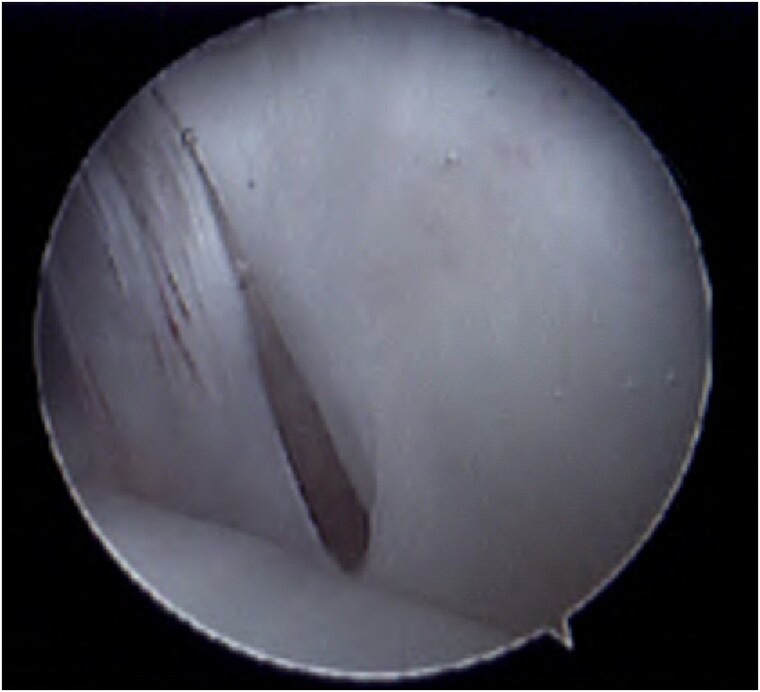
Intra-operative picture demonstrating congenitally absent bicipital groove.

At 3 months postoperatively, he had full range of motion and shoulder strength, reporting significantly improved pain. At this time point he transitioned to a home exercise program to follow-up as needed.

## Discussion

Previous studies have postulated the LHBT function as a dynamic depressor of the humeral head, affecting glenohumeral joint stability [6–8]. Patients with this congenital absence commonly present with shoulder instability and/or impingement signs [2, 3]. Interestingly, a previous EMG study demonstrated the LHBT only playing a significant role in glenohumeral joint stabilization in the unstable shoulder implying its role as a secondary stabilizer [9]. The significance of the LHBT function comes into question mainly from previous case reports indicating absence without major instability or impingement [10, 11].

Clinical identification of a congenitally absent LHBT can be near impossible due to the range of possible differential diagnoses for non-specific shoulder pain, instability, or impingement, including diagnoses of rotator cuff pathology, biceps muscle and tendon pathology, and labral pathology. Evaluation includes examination for indications inferring typical pathology such as Popeye’s sign indicating possible proximal biceps rupture, weakness with Jobe test indicating possible rotator cuff pathology, or other specialty tests such as O’Brien, Speed, and Yergason testing. Gold standard for diagnosis is shoulder arthroscopy. Key imaging indicators include a smooth, shallow intertubercular groove and absence of a torn or retracted biceps tendon [12].

The LHBT originates during early embryonic development between 6 and 8 weeks from mesoderm of the arm bud [13], the same anlage that later gives rise to the glenoid, labrum, and capsule [14]. Visualized as a discrete cord passing over the humeral head and attaching to the glenoid labrum at 9 weeks, it becomes more flattened between weeks 23 and 40; by adulthood it becomes triangular in shape [13, 15]. Anomalies may arise from disruptions during early development [3]. Previous reports have identified a link between congenital absence of the LHBT and other congenital abnormalities, including VATER syndrome and spina bifida [4, 16, 17].

In one systematic review analysing 35 previously reported cases [2], only 13 were complete absences of LHBT. The most common presenting symptoms were shoulder pain (85.7%), followed by shoulder instability (37.1%), and impingement (25.7%). Interestingly, among patients with instability, only one had posterior instability. Most presentations were non-traumatic; only two patients presented post-traumatic injury [14, 18]. Amongst MRI findings in 25/35 cases, 60% initially failed to identify the absence. In these instances, absence of the LHBT was detected during shoulder arthroscopy and later confirmed through retrospective MRI analysis. Our patient was 21 years old at detection, whereas the average age of detection in the systematic review was 33 years; only six patients were 21 years old or younger, all presenting with pain and some degree of instability. The majority experienced significant symptom improvement or complete resolution within three months after treatment. Of available long-term follow-ups, patients remained almost symptom-free, with little to no pain or instability for over a year [3, 5, 19–22].

Since the publication of the systematic review in 2017, six additional cases of complete congenital absence of the LHBT have been reported. Of these, only one case presented following a post-traumatic injury [23]. Five of the six cases presented with shoulder pain, while four exhibited instability [11, 16, 23–25]. One further supports the association between congenital anomalies and absent LHBT, describing a patient with Silver–Russell syndrome found to have bilateral congenital absence of the LHBT [25]. Only one case involved a patient younger than 21 years old [24].

The case presented is unique because the patient was found to have a congenital absence of the LHBT following trauma, only the third known reported in this setting without other histories of prior shoulder pain or instability and is one of the few cases of congenitally absent LHBT in which the condition was initially detected on MRI prior to arthroscopy.

## Conclusion

Congenitally absent LHBT may not always present with prior shoulder pain or instability; evidence of abnormality on MRI prior to diagnostic arthroscopy confirmation may aid diagnosis.
